# MicroRNA-related polymorphisms in apoptosis pathway genes are predictive of clinical outcome in patients with limited disease small cell lung cancer

**DOI:** 10.18632/oncotarget.8134

**Published:** 2016-03-16

**Authors:** Wei Jiang, Nan Bi, Wen-Jue Zhang, Li-Hong Wu, Li-Pin Liu, Yu Men, Jing-Bo Wang, Jun Liang, Zhou-Guang Hui, Zong-Mei Zhou, Lu-Hua Wang

**Affiliations:** ^1^ Department of Radiation Oncology, Cancer Hospital, Chinese Academy of Medical Sciences and Peking Union Medical College, Beijing, China

**Keywords:** small cell lung cancer, limited-disease, single nucleotide polymorphisms, microRNA, apoptosis pathway

## Abstract

We examined the impact of single nucleotide polymorphisms (SNPs) at miRNA binding sites in the 3′-UTRs of genes in the apoptosis pathway on the prognosis of patients with limited disease-small cell lung cancer (LD-SCLC). Twelve tagSNPs in seven genes were genotyped using blood samples from 146 LD-SCLC patients treated with chemoradiotherapy. Cox proportional hazard regression models and recursive partitioning analysis were performed to identify SNPs significantly associated with overall survival. Three SNPs, *CASP8:* rs1045494 (C > T), *PIK3R1:* rs3756668 (A > G) and *CASP7:* rs4353229 (T > C), were associated with longer overall survival in LD-SCLC patients after chemoradiotherapy. The adjusted hazard ratios (95% confidence intervals) were 0.480 (0.258–0.894), 0.405 (0.173–0.947) and 0.446 (0.247–0.802), respectively, and remained significant after multiple comparison correction. Moreover, subset analysis showed these SNPs were still predictive of overall survival in stage III patients. Recursive partitioning analysis enabled patients to be classified into three risk subgroups based on unfavorable genotype combinations of the rs1045494 and rs4353229 SNPs. These findings suggest miRNA-related polymorphisms in the apoptosis pathway may be useful biomarkers for selection of LD-SCLC patients likely to benefit from chemoradiotherapy.

## INTRODUCTION

Small cell lung cancer (SCLC), which accounts for approximately 15% of primary lung cancers [1], is a very aggressive neuroendocrine malignancy characterized by early metastasis and a poor prognosis. Combined chemoradiotherapy (CRT) is administered to the one-third of SCLC patients who present with limited disease (LD-SCLC) at diagnosis [2]. However, their prognosis remains poor. There is also substantial inter-individual variation in the clinical response to treatment, which suggests genetic variation plays an important role in determining patients likely to benefit from CRT.

SCLC is characterized by a short doubling time and fast growth, and almost no apoptotic cells are detected in tumor specimens [3]. Cisplatin-based regimens are the standard chemotherapy for SCLC. Cisplatin would be expected to activate apoptotic signaling and kill cells. Likewise, radiotherapy also works by damaging DNA and inducing apoptosis [4]. Altered apoptotic signaling is thought to contribute to tumorigenesis and progression [5], and genetic variants in the apoptotic pathway are reportedly associated with prognosis and treatment strategy in several cancers [5–7], including SCLC [8]. MicroRNAs (miRNAs), a class of small noncoding RNAs, are key regulators of gene expression in many biological processes [9]. Single nucleotide polymorphisms (SNPs) located in the 3′-UTR of miRNA target genes may affect the actions of miRNAs at oncogenes and tumor suppressor genes [10]. However, few published studies have considered the impact of SNPs at miRNA-binding sites in apoptosis pathways and their relationship to SCLC outcomes. We therefore analyzed the effect of variations at miRNA-binding sites in apoptosis pathways on the prognosis of LD-SCLC patients.

## RESULTS

### Clinical characteristics

Ultimately, 146 patients who received curative chemoradiotherapy for LD-SCLC were included in the study. Of those, 91.8% were diagnosed at stage III. EP (etoposide + cisplatin) and EC (etoposide + carboplatin) were the most common regimens. Chemotherapy was delivered concurrently or sequentially. Prophylactic cranial irradiation (PCI) was administered to 43.2% of patients. In 90.5% of those, the primary lesion showed a complete remission (CR) or a partial response (PR). The overall median survival time (MST) and 5-year overall survival (OS) rate were 35.1 months and 38.9%, respectively. The median follow up time was 42.2 months. Age, Karnofsky performance score (KPS), Charison comorbidity index (CCI) and PCI were all significantly associated with the survival outcome (*p* < 0.05; log-rank test). The clinical characteristics of the patients are summarized in Table 1.

**Table 1:** Clinical characteristics of patients with limited disease-small cell lung cancer

Variables		*N* (%)	5y OS	*P*
Gender	Male	104 (71.2%)	33.00%	0.065
	Female	42 (28.8%)	47.20%	
Age	≤ 60	95 (65.1%)	43.00%	0.015
	> 60	51 (34.9%)	30.80%	
KPS	≥ 90	61 (41.8%)	47.50%	0.009
	< 90	85 (58.2%)	33.10%	
Location	Left lobe	69 (47.3%)	39.30%	0.987
	Right lobe	77 (52.7%)	37.80%	
Smoking	Yes	96 (65.8%)	35.70%	0.088
	No	50 (34.2%)	40.10%	
Charison comorbidity index	≤ 3	130 (89.0%)	41.70%	0.03
	4–5	13 (8.9%)	19.50%	
	6–7	3 (2.1%)	0%	
Weight loss	With	27 (18.5%)	51.30%	0.393
	Without	119 (81.5%)	35.20%	
AJCC stage	I A	1 (0.7%)	100.00%	0.573
	I B	2 (1.4%)	0%	
	II A	5 (3.4%)	NA	
	II B	4 (2.7%)	NA	
	III A	68 (46.6%)	41.70%	
	III B	66 (45.2%)	34.90%	
Treatment modality	Concurrent	80 (54.8%)	46.80%	0.401
	Sequential	66 (45.2%)	31.20%	
Chemotherapy cycles	< 4	9 (6.2%)	NA	0.382
	4–6	126 (86.3%)	40.20%	
	> 6	11 (7.5%)	38.60%	
Radiotherapy dose	< 60	46 (30.7%)	39.30%	0.525
	≥ 60	100 (69.3%)	39.90%	
PCI	With	63 (43.2%)	62.10%	1.54E-04
	Without	83 (56.8%)	23.10%	

Abbreviations: OS, Overall Survival; AJCC, American Joint Committee on Cancer; PCI, Propylactic Cranial Irradiation.

### Association of individual SNPs with overall survival

**Figure 1:**
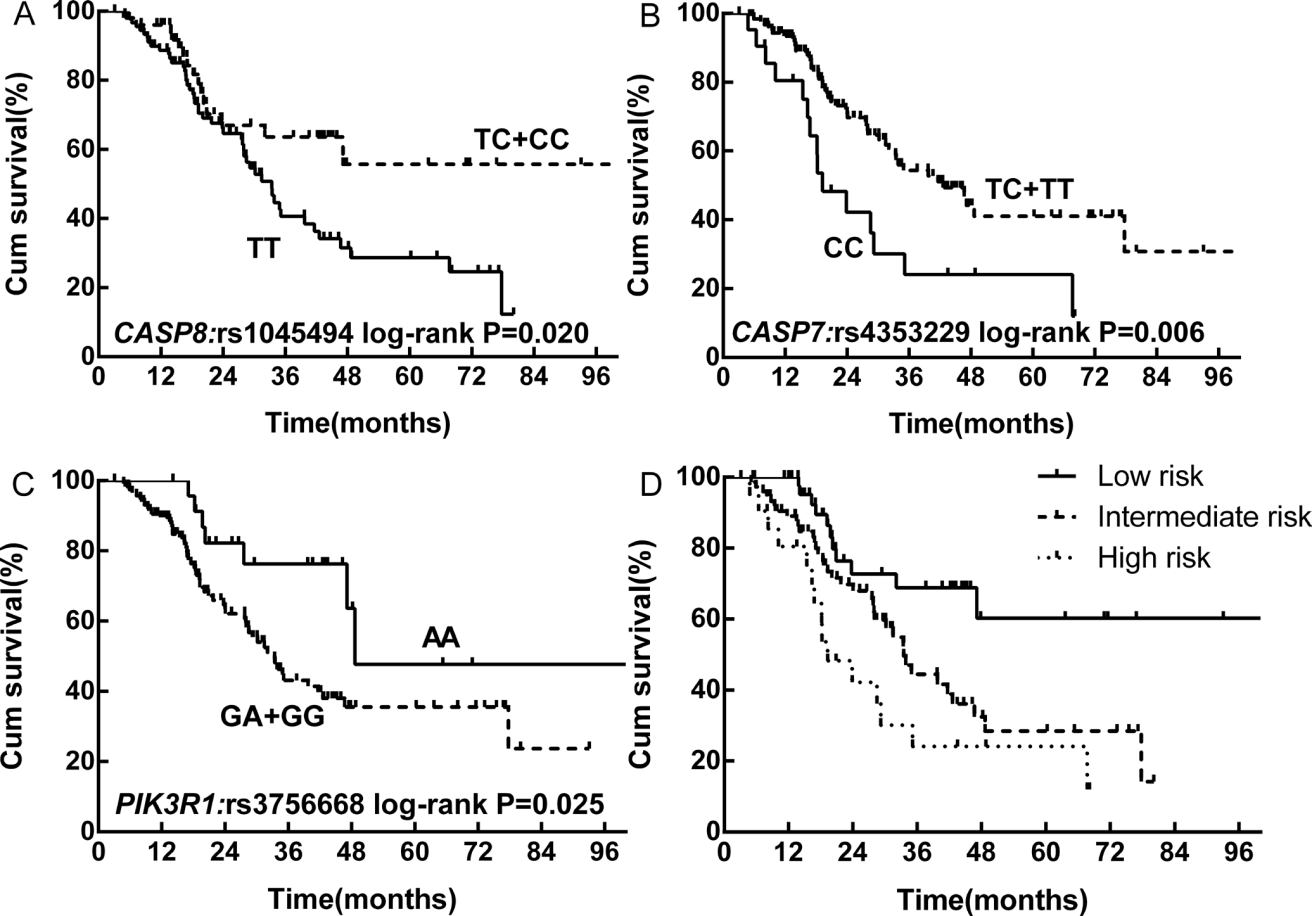
Kaplan-Meier survival curves for selected SNPs and the combined RPA classification in patients with LD-SCLC treated with curative chemoradiotherapy (**A**) *CAP8:* rs1045494. (**B**) *CASP7:* rs4353229. (**C**) *PIK3R1:* rs3756668. (**D**) RPA classification.

Three SNPs were significantly associated with OS, and the association remained significant after adjusting for age, gender, KPS, smoking history, CCI and PCI (Table 2). One variant of *CASP8* (HGNC:1509), rs1045494, had a 48.6% lower risk of death (adjusted hazard ratio [HR] = 0.514, 95% confidence interval [CI]: 0.287–0.921). In addition, the variant homozygous genotype of rs4353229 in *CASP7* (HGNC:1508) was associated with a 2.245-fold increase in the risk of death (adjusted HR = 2.245, 95% CI: 1.247–4.041), while rs3756668 in phosphoinositide-3-kinase regulatory subunit 1 (alpha) (*PIK3R1*, HGNC:8979) was associated with a 57.9% decrease in the risk of death (adjusted HR = 0.421, 95% CI: 0.190–0.931) (Figure 1A–1C). After multiple comparison correction, these SNPs still had a *q* < 0.05. Two other SNPs, rs12755 in *PIK3R1* and rs13429049 in BCL2-like 11 (apoptosis facilitator) (*BCL2L11*, HGNC:994), were also significantly associated with OS (Supplementary Table S1).

**Table 2:** Survival analysis of miRNA-related SNPs in apoptosis pathway genes

Gene SNP	miRNA	Model		5y OS	HR (95% CI)	Log-rank *P*	HR (95%CI) (adjusted) ^*^	*P* adjusted	*q*
*CASP8* rs1045494		DOM	TT (90)	28.70%	0.508 (0.284–0.909)	0.02	0.514 (0.287–0.921)	0.025	0.035
			TC+CC (53)	55.70%					
*PIK3R1* rs3756668	miR-589–3p	REC	GA+GG (119)	35.50%	0.417 (0.190–0.916)	0.025	0.421 (0.190–0.931)	0.033	0.037
			AA (25)	47.70%					
*CASP7* rs4353229	miR-520a-5p	REC	TC+TT (125)	41.00%	2.227 (1.246–3.980)	0.006	2.245 (1.247–4.041)	0.007	0.023
			CC (21)	24.10%					

* Adjusted for age, gender, Karnofsky performance score (KPS), smoking history, Charison comorbidity index (CCI) and propylactic cranial irradiation (PCI).

Abbreviations: SNP, single nucleotide polymorphism; OS, overall survival; HR: hazard ratio; CI, confidence interval.

### Recursive partitioning analysis (RPA)

Five SNPs significantly associated with OS after multiple comparisons (*q* < 0.05) (rs1045494 in a dominant model and rs12755, rs13429049, rs3756668 and rs4353229 in recessive models) were included in a recursive partitioning analysis of the effect of unfavorable genotype combinations. Rs1045494 and rs4353229 were eventually selected to develop a RPA model that enabled us to split the data set into three risk classifications (Table 3). The 5-year OS rates were 60.3%, 28.4% and 24.1% in the low-, intermediate- and high-risk classes, respectively (Figure 1D).

**Table 3:** RPA classification based on unfavorable genotype combinations

	rs 4353229	rs1045494	*n*	5y-OS	HR (95% CI) adjusted	*P* adjusted^*^	*P* bootstrap
Low risk	TT+TC	CC+TC	48	60.3%	Ref	0.005	
Intermediate risk	TT+TC	TT	74	28.4%	2.150 (1.081–4.275)	0.029	0.036
High risk	CC	Any	21	24.1%	3.760 (1.680–8.415)	0.001	0.007

* Adjusted for age, gender, Karnofsky performance score (KPS), smoking history, Charison comorbidity index (CCI) and propylactic cranial irradiation (PCI).

Abbreviations: RPA, recursive partitioning analysis; OS, overall survival; HR, hazard ratio; CI: confidence interval.

### Association of individual SNPs with OS in stage III patients

In the 134 patients with stage III SCLC, the overall MST and 5-year OS rate were 35.1 months and 38.2%, respectively. Rs1045494, rs3756668 and rs4353229 remained the three SNPs associated with survival (Supplementary Table S2). The associated HRs adjusted for clinical covariates were 0.480, 0.405 and 2.316, respectively. After correcting for multiple comparisons, genetic variants of rs1045494 and rs4353229 continued to exhibit significant prognostic value, while the association of rs3756668 was borderline.

### Validation of the RPA classification using bootstrap analysis

The RPA predictive model based on SNPs in the apoptosis pathway was internally validated using bootstrap analysis with a 1000-resample set. The differences in OS among the RPA classes were significant (*p* < 0.0001; log-rank test). The concordance probability estimate (CPE) used to quantify the RPA classification's discrimination demonstrated good performance in the resampling internal validation (0.71) datasets.

## DISCUSSION

Apoptosis, or programmed cell death, is a fundamental process of controlled cell elimination essential for cellular homeostasis in multicellular organisms [11]. There are two principal interconnected signaling pathways that respectively control apoptosis by activating caspases via the *Bcl-2* family and death receptor-related factors [12]. Expression of variants of the genes involved can lead to apoptotic dysregulation that contributes to the development of cancer [13] or is associated with cancer susceptibility and progression [14–16].

In this study, we identified three SNPs at miRNA binding sites on target genes in the apoptosis pathway that affected the OS of LD-SCLC as well as stage III SCLC patients who underwent curative chemoradiotherapy. Moreover, the unfavorable genotype combination of *CASP8*: rs1045494 and *CASP7*: rs4353229 amplified the effect of the individual polymorphisms and were of greater potential predictive value for LD-SCLC patients. This is the first study evaluating the combined effect of polymorphisms interacting with apoptosis in SCLC. Through multigenic variant combination, these patients could be classified into three risk groups, which provided a basis to identify LD-SCLC patients who would benefit from chemoradiotherapy.

*CASP7*: rs4353229 was the most significant SNP associated with survival in these patients. Caspases are a family of cysteine-aspartic acid proteases that serve as initiators or executioners in cell apoptosis [17]. The *CASP7* gene, located in exon 21 of chromosome 10q25, plays a crucial role during the execution phase of apoptosis. The loss of its expression is reportedly associated with a poor prognosis in renal cell carcinoma [18]. The *CASP7* rs4353229 SNP was also determined to be predictive of survival in patients with advanced non-small cell lung cancer [19]. However, there has been no direct functional study of rs4353229 variants. Instead, possible underlying mechanisms were inferred from data publically available online [20]. Rs4353229 C allele was reported to be associated slightly decreased levels of RNA expression in Asian population. On the other hand, the rs4353229 SNP is strongly linked to disequilibrium with the *CASP7* rs2227309 SNP in the Chinese population (*r*2 = 0.98; D’ = 1.0), which is thought to influence the level of *CASP7* mRNA expression and individuals' apoptotic capacity [21].

The *CASP8*: rs1045494 SNP was previously reported to be associated with breast cancer risk [22]. Caspase 8 is an apoptosis initiator in the extrinsic cell death pathway mediated by tumor necrosis factor (TNF) [23]. Soung et al. [24] demonstrated that somatic mutations decrease *CASP-8* activity during apoptosis as compared with wild-type. Notably, caspase-8 expression is reportedly absent in SCLC, leading SCLC cells to be resistant to apoptosis [25, 26]. In our study, the rs1045494 C variant was significantly associated with a much better outcome than wild-type, suggesting the polymorphisms in the miRNA binding site enhanced the expression of caspase-8. Confirmation of the mechanism requires further investigation, however.

The A/A genotype of rs3756668, located in the 3′-UTR of *PIK3R1*, was associated with a considerable improvement in survival, though it did not enter the combined RPA model. *PIK3R1* is a component of the phosphoinositide-3 kinase (*PI3K*)-*AKT*-mammalian target of rapamycin (*mTOR*) pathway and encodes the 85-kD regulatory subunit of *PI3K*. *In vitro* molecular investigation revealed that heterozygous disruption of *PIK3R1* decreased apoptosis induced by insulin-like growth factor 1 (*IGF-1*) through up-regulated phosphatidylinositol (3, 4, 5)-triphosphate production, while homozygous *PIK3R1* significantly increased apoptosis reflecting a reduction in PI3-kinase activity. This is consistent with our analysis of the rs3756668 genotype (Supplementary Table S1) and suggests an effect of this SNP on *PIK3R1* expression.

This is the first report on the association of SNPs affecting miRNA binding to targets in the apoptosis pathway on clinical outcome in SCLC. The adoption of a targeted pathway-based approach strengthens the effective power of the study and provides greater discrimination of outcomes with multigenic effects. Within the apoptotic signaling network, activated caspase-8 cleaves caspase-3 and/or caspase-7, initiating the apoptotic cascade [27]. Consistent with that, our findings show that the combined effect of the two SNPs in *CASP8* and *CASP7* is greater than that of the individual SNPs. However, the *PIK3R1* SNP in another pathway could not be included in the final prognostic model. This may be due to the small number of SNPs selected in this in silico analysis, which potentially missed some key sites of interaction between different pathways and is a limitation of this study. Another limitation is that although this is the largest study yet conducted to explore miRNA-related SNPs associated with prognosis in SCLC, we did not recruit enough cases for independent validation. Finally, although evidence from previous studies support the biological plausibility of our findings, *in vitro* functional assays of the identified SNPs will be necessary determine the molecular mechanisms.

In sum, the present study showed that three SNPs affecting miRNA binding sites on components of the apoptosis pathway are prognostic in LD-SCLC patients treated with chemoradiotherapy. Moreover, a risk classification incorporating *CASP8*: rs1045494 and *CASP7*: rs4353229 was developed to identify patients likely to benefit from treatment. Replication in large independent cohorts and determination of the molecular mechanism are imperative to confirm these findings.

## MATERIALS AND METHODS

### Ethics statement

This investigation was conducted in accordance with the ethical standards outlined in the Declaration of Helsinki and national and international guidelines. Our institutional review board (Cancer Hospital, Chinese Academy of Medical Sciences) approved this retrospective study and informed consent was waived.

### Study population

Patients histologically confirmed as SCLC and initially treated at the Cancer Hospital, Chinese Academy of Medical Sciences between January 2007 and June 2014 were enrolled in this study. The inclusion criteria were as follows: (1) LD-SCLC staged based on the International Association of Lung Cancer (IASLC) classification [28]; (2) patients received curative-intent platin-based chemotherapy combined intensity-modulated radiation therapy (IMRT). Patients who had any other malignancy within 5 years of enrollment were excluded. Clinical information was collected from medical records. Long-term archived serum samples from these patients were analyzed [29].

### SNP selection and genotyping

Candidate SNPs at miRNA-binding sites in the 3′-UTR of apoptotic pathway genes were selected using the PolymiRTS Database 3.0 (http://compbio.uthsc.edu/miRSNP/) and Ensemble Asia database (release 79, http://asia.ensembl.org/index.html?redirect = no). SNPs in the apoptotic pathway previously reported in the literature to be associated with cancer were also included. Tagged SNPs in linkage disequilibrium were identified using HaploReg version 3 (http://www.broadinstitute.org/mammals/haploreg/haploreg_v3.php) with a cut-off value of *r*^2^ > 0.8 and a minor allele frequency greater than 0.01 in the Chinese Han population.

DNA was extracted using a TIANamp Blood DNA Kit (TIANGEN BIOTECH, Beijing, China). Genotyping was conducted using a MALDI-TOF mass spectrometry-based iPLEX Gold assay on a Sequenom MassARRAY Platform (San Diego, CA, USA), and was analyzed using MassARRAY TyperAnalyzer v4.0 software (Sequenom). SNPs with a success rate of more than 95% and samples with a call rate exceeding 96% were included. Ultimately, a total of 12 tagSNPs in 7 genes were selected.

### Statistical analysis

To ensure the reliability of the genotyping, the Hardy-Weinberg equilibrium was tested using the chi-square goodness-of-fit test with one degree of freedom. OS was calculated from pathologic diagnosis to the date of death or last follow-up. The survival estimates for each genotype and for clinical characteristics were calculated using the Kaplan-Meier method and compared using the log rank test. Hazard ratios (HRs) and 95% confident intervals (CIs) were estimated using Cox proportional hazards regression models, adjusting for clinical characteristics associated with OS. All SNPs were analyzed in three genetic models (dominant, recessive and additive), and the most significant model was used. Models with a rare genotype observed < 5% percent of patients were excluded. Benjamini-Hochberg False Discovery Rate (FDR) correction was used for multiple testing with a *q*-value of 0.05 [30]. To evaluate the cumulative effects of the genetic variants in the pathway, recursive partitioning analysis (RPA) was conducted to combine the prognostic SNPs. The concordance probability estimate (CPE) was used to assess the predictive ability of the RPA classification [31] from 1000 bootstrap samples.

A two-sided *p* values < 0.05 was considered significant for all the statistical analyses. All the statistical analyses were carried out using IBM SPSS Statistics 21.0 software (IBM Corp., Armonk, NY) and R version 3.1.3 (www.R-project.org).

## SUPPLEMENTARY MATERIALS TABLES


