# Supplementary material for: MicroRNA-related polymorphisms in apoptosis pathway genes are predictive of clinical outcome in patients with limited disease small cell lung cancer
Source: Oncotarget. 2016 Mar 16;7(16):22632–8. doi: 10.18632/oncotarget.8134 (PMC5008387; doi:10.18632/oncotarget.8134)
Supplement: Supplementary file 1 [file oncotarget-07-22632-s001.pdf]

MicroRNA-related polymorphisms in apoptosis pathway genes are predictive of clinical outcome in patients with limited disease small cell lung cancer

Supplementary Materials

Supplementary Table S1: Survival analysis of miRSNPs in apoptostic pathway in three models in 146 patients with limited-disease small cell lung cancer

Gene SNP	miRNA	Model		5yOS	HR (95% CI)	Log-rank *P*	HR (95% CI) (adjusted)	*P* adjusted	*q*
BIRC5 rs1042542		DOM	CC (59)	44.2%	1.208 (0.725–2.010)	0.467	1.402 (0.838–2.345)	0.198	0.086
			CT+TT (86)	35.8%					
CASP8 rs1045494		DOM	TT (90)	28.7%	0.508 (0.284–0.909)	0.020	0.514 (0.287–0.921)	0.025	0.035
			TC+CC (53)	55.7%					
CASP7 rs1127687	miR-140–5p	DOM	GG (92)	40.3%	1.146 (0.685–1.917)	0.603	1.411 (0.836–2.381)	0.197	0.086
			GA+AA (52)	35.2%					
CASP7 rs12247479	miR-1260b	DOM	GG (113)	35.7%	0.632 (0.329–1.213)	0.164	0.654 (0.337–1.269)	0.209	0.088
			GA+AA (33)	51.3%					
PIK3R1 rs12755	miR-154–3p	REC	CA+CC (134)	36.8%	0.332 (0.081–1.361)	0.107	0.271 (0.065–1.124)	0.072	0.049
			AA (11)	71.1%					
BCL2L11 rs13429049	miR-548n	REC	CT+CC (134)	NA	0.340 (0.083–1.396)	0.116	0.202 (0.039–1.038)	0.055	0.045
			TT (10)	36.7%					
BCL2 rs1564483	miR-296–3p	DOM	CC (69)	50.5%	1.395 (0.836–2.328)	0.201	1.303 (0.765–2.219)	0.330	0.116
			CT+TT (74)	31.5%					
PIK3R1 rs3756668	miR-589–3p	REC	GA+GG (119)	35.5%	0.417 (0.190–0.916)	0.025	0.421 (0.190–0.931)	0.033	0.037
			AA (25)	47.7%					
CASP7 rs4353229	miR-520a-5p	REC	TC+TT (125)	41.0%	2.227 (1.246–3.980)	0.006	2.245 (1.247–4.041)	0.007	0.024
			CC (21)	24.1%					
TP53 rs4968187	miR-4716–5p	ADD	CC (131)	39.2%	1.051 (0.419–2.635)	0.915	0.901 (0.351–2.314)	0.828	0.245
			CT (13)	44.3%					

BCL2L11 rs6753785	miR-556–5p	DOM	GG (65)	46.6%	1.517 (0.914–2.518)	0.104	1.293 (0.757–2.209	0.346	0.119
			GT+TT (80)	31.7%					
BCL2L11 rs72837819	miR-24–1–5p	DOM	AA (88)	35.7%	0.718 (0.419–1.231)	0.227	1.096 (0.620–1.937)	0.752	0.227
			AG+GG (55)	46.5%					

Supplementary Table S2: Survival analysis of miRSNPs in apoptostic pathway in three models in 133 patients with stage III small cell lung cancer

Gene SNP	miRNA	Model		5yOS	HR (95% CI)	Log-rank *P*	HR (95% CI) (adjusted)	*P* adjusted	*q*
BIRC5 rs1042542		DOM	CC (52)	40.0%	1.081 (0.635–1.840)	0.733	1.388 (0.795–2.421)	0.248	0.123
			CT+TT (81)	37.3%					
CASP8 rs1045494		DOM	TT (82)	26.6%	0.442 (0.238–0.821)	0.008	0.480 (0.258–0.894)	0.021	0.042
			TC+CC (49)	57.4%					
CASP7 rs1127687	miR-140–5p	DOM	GG (83)	39.7%	1.165 (0.684–1.985)	0.574	1.471 (0.856–2.528)	0.162	0.093
			GA+AA (49)	34.2%					
CASP7 rs12247479	miR-1260b	DOM	GG (107)	34.9%	0.559 (0.265–1.183)	0.123	0.536 (0.250–1.146)	0.108	0.072
			GA+AA (27)	53.7%					
PIK3R1 rs12755	miR-154–3p	REC	CA+CC (123)	36.2%	0.360 (0.088–1.475)	0.138	0.290 (0.070–1.208)	0.089	0.069
			AA (10)	70.0%					
BCL2L11 rs13429049	miR-548n	REC	CT+CC (122)	NA	0.334 (0.081–1.374)	0.110	0.230 (0.044–1.205)	0.082	0.067
			TT (10)	35.8%					
BCL2 rs1564483	miR-296–3p	DOM	CC (66)	48.7%	1.288 (0.759–2.186)	0.347	1.146 (0.658–1.996)	0.630	0.240
			CT+TT (65)	31.1%					
PIK3R1 rs3756668	miR-589–3p	REC	GA+GG (109)	34.0%	0.362 (0.155–0.845)	0.014	0.405 (0.173–0.947)	0.037	0.049
			AA (23)	49.5%					
CASP7 rs4353229	miR-520a-5p	REC	TC+TT (113)	40.5%	2.239 (1.243–4.033)	0.006	2.316 (1.272–4.215)	0.006	0.024
			CC (21)	24.1%					
TP53 rs4968187	miR-4716–5p	ADD	CC (121)	38.2%	1.116 (0.401–3.108)	0.834	0.881 (0.304–2.555)	0.816	0.290
			CT (11)	53.0%					

BCL2L11 rs6753785	miR-556–5p	DOM	GG (60)	45.6%	1.559 (0.919–2.644)	0.097	1.326 (0.757–2.324)	0.324	0.144
			GT+TT (73)	31.5%					
BCL2L11 rs72837819	miR-24–1–5p	DOM	AA (79)	35.7%	0.786 (0.453–1.364)	0.391	1.249 (0.694–2.249)	0.458	0.187
			AG+GG (52)	44.5%					
